# The antimethanogenic efficacy and fate of bromoform and its transformation products in rumen fluid

**DOI:** 10.1038/s41598-025-10936-9

**Published:** 2025-07-11

**Authors:** Kevin M. Posman, Gabriella Iacono, Carmen M. Cartisano, Sarah Y. Morrison, David Emerson, Nichole N. Price, Stephen D. Archer

**Affiliations:** 1 Bigelow Laboratory for Ocean Sciences, 60 Bigelow Drive, East Boothbay, Maine, 04544 USA; 2 The William H. Miner Agricultural Research Institute, 1034 Miner Farm Road, Chazy, NY 12921 USA; 3 Environmental Studies, Colby College, 4000 Mayflower Hill Dr, Waterville, ME 04901 USA

**Keywords:** Methane, Bromoform, Dibromomethane, Dehalogenation, Rumen, Algae, Seaweed, Biochemistry, Microbiology, Animal physiology

## Abstract

Enteric methane emissions from ruminant livestock are a significant source of atmospheric methane. Efforts to address rising atmospheric methane concentrations have led to an expansion of research into mitigating enteric methane production. One of the most effective approaches utilizes bromoform-containing feed supplements, such as the algae *Asparagopsis* spp., to inhibit methanogenesis in the rumen. Understanding the fate and persistence of bromoform in the rumen is important for developing safe, effective products and feeding strategies. This study conducted a series of in vitro rumen fluid experiments monitoring bromoform, dibromomethane, and bromomethane concentrations, methane production and several biochemical parameters to understand the inhibitory thresholds and degradation processes of these compounds. Analysis of the rumen fluid confirmed bromoform is rapidly dehalogenated. The half-life of bromoform was 26 min, coinciding with the production of dibromomethane accumulating to 22.1% of the initial bromoform amendment, but no bromomethane was detected. Dibromomethane demonstrated a considerably longer half-life of 775 min. In separate dose-response experiments, bromoform, dibromomethane and bromomethane all exhibited anti-methanogenic activity. Bromoform and dibromomethane produced sigmoidal-relationships between concentration and inhibition at approximately 1–2 µM, and yielded similar effective concentration values (EC50s) for antimethanogenic activity. Experiments using *Asparagopsis taxiformis* algae revealed less accumulation of bromoform and formation of dibromomethane, likely driven by a slower release from the seaweed material. The *A. taxiformis* dose response was less effective at inhibiting methane per mole of bromoform added compared with direct bromoform additions. These results have significant implications for understanding the dynamics of bromoform-mediated methane inhibition and will aid the development of effective halocarbon additives, feeding strategies, and testing protocols for bromoform and its degradation byproducts.

**Supplementary Information:**

The online version contains supplementary material available at 10.1038/s41598-025-10936-9.

## Introduction

Addressing enteric methane (CH_4_) emissions is a priority for lowering the carbon intensity of livestock agriculture^1^. Strategies to reduce livestock CH_4_ emissions include improved diet quality, breeding more efficient animals, developing antibiotics and vaccines to reduce rumen methanogen abundance and feed supplements that inhibit methanogenesis. While breeding and feed quality have driven incremental improvements in CH_4_ production metrics e.g. CH_4_ yield (g CH_4_/kg dry matter intake (DMI)), CH_4_ intensity (g CH_4_/kg milk or beef produced) and CH_4_ production (g CH_4_/d), a number of feed additives have proven particularly effective and are starting to be produced at commercial scale (reviewed in^2,3^. Integration of these formulations into existing feed manufacturing and farm operations could be an important tool for rapidly addressing environmental concerns for dairy and beef production in particular.

The advantages of feed additives are the potential for fast-acting and significant reductions in enteric CH_4_ production. Multiple studies, using either in vitro or in vivo approaches, have examined the efficacy of the variety of antimethanogenic feed additives. A meta-analysis of in vitro studies showed that the mean reduction in CH_4_ production among 14 categories of feed additive from 277 studies, ranged from − 75% to + 5%, with a median value of −17%^4^. Similarly, a meta-analysis of in vivo studies of the impact of feed additives on CH_4_ production, indicated a similar range of effect size, from − 70% to + 5% in CH_4_ yield; with the maximum suppression, averaging − 49%, associated with seaweed products^5^. Among seaweed species, maximum reductions of 67–98% in CH_4_ production, have been demonstrated when feeding the red macroalgae genus *Asparagopsis* (*A. taxiformis* and *A. armata*), to sheep^6^, beef cattle^7,8^ and dairy cattle^9,10^. The antimethanogenic bioactive ingredients in *Asparagopsis* are a suite of halogenated compounds, dominated by bromoform (CHBr_3_)^11–13^. Variability in the levels of CH_4_ reduction within and between experimental studies, can generally be attributed to the amount of bromoform intake (e.g. mg CHBr_3_/kg DMI), reflecting the direct impact of these compounds on methanogenesis in the rumen^14^. Halogenated aliphatic compounds that have been shown to effectively reduce enteric CH_4_ emissions in animal studies are generally polyhalogenated C1 compounds and include chloroform in sheep^15^ and dairy cows^16^; bromochloromethane in beef cattle^17^ and goats^18,19^; bromoform in dairy and beef cattle (e.g.^20,21^); and iodoform in dairy cows^22^.

The reduction of CO_2_ or formate to CH_4_ by hydrogenotrophic methanogenic archaea dominates CH_4_ production in the rumen, with relatively minor contributions from methylotrophic pathways using methanol and methylamines as substrates and via the aceticlastic pathway that cleaves acetate to generate the required methyl group^23^. The three pathways of CH_4_ production share two final enzymatic steps. These involve coenzyme M methyltransferase complex (Mtr), which catalyzes the transfer of a methyl group to coenzyme M; and methyl-coenzyme M reductase (Mcr) which then reduces the methyl group to CH_4_. Although the mechanisms are not fully understood, halogenated C1 compounds, or halomethanes, may competitively inhibit both the Mtr-catalyzed and Mcr-catalyzed steps in methanogenesis^24,25^. Inhibition of the methanogenesis with halomethanes has been demonstrated to reduce the abundance of the methanogen population both in vitro^12^ and in vivo^19,26,27^, correlating with the decrease in CH_4_ production.

Although inclusion of halomethanes in the diet of ruminants is highly effective at reducing CH_4_ production, the possible transfer of halogenated compounds to animal tissues and products or subsequent release into the environment raises health and environmental concerns (reviewed in Glasson et al.^28^). Investigation of the toxicity of halomethanes, particularly trihalomethanes (THMs) such as bromoform, has been stimulated primarily by concerns over the extensive use of chlorination to disinfect drinking water that generates a variety of THMs as byproducts. There is evidence that THMs have cytotoxic, genotoxic and mutagenic effects, although little is known about the risks to human health^29^. The composition of THMs also influences the short and long-term toxicological risk, with greater cytotoxic and mutagenic effects associated with brominated versus chlorinated compounds^29,30^. A thorough understanding of the transformation and fate of halogenated compounds in livestock feed additives is clearly required.

In general, studies that have fed *Asparagopsis* directly or in the form of oil-extracts to ruminants at effective inclusion levels to reduce CH_4_ production have found minimal accumulation of bromoform in milk, tissue, urine or fecal matter^9,20,21^. Studies that used higher inclusion levels did detect bromoform in the milk and urine^10,31^, though inconsistently and generally at concentrations well below guidelines for safe levels for drinking water advised by regulatory bodies such as the World Health Organization, European Union, United States Environment Protection Agency and Health Canada^32^.

Metabolism of bromoform and similar halomethanes by microbes in the rumen may be partly responsible for the low concentrations absorbed, transferred to milk and tissue or excreted by livestock. Reductive dehalogenation of halomethanes by methanogenic microbes has been linked to the activity of Mtr and Mcr enzymes involved in methanogenesis, with the latter complex considerably more effective as a degradation catalyst^24^. Microbial dehalogenation of *Asparagopsis*-derived bromoform has recently been demonstrated in in vitro assays of rumen microbes and in pure cultures of a variety of methanogen strains^33^. Understanding the degradation processes of bromoform and related compounds in the rumen is critical to developing optimal dose formulations of halomethane-containing feed additives. Optimal dosing of bromoform has environmental, economic and management implications throughout dairy and beef supply chains, including reducing costs and potentially increasing adoption of CH_4_-mitigation strategies^21^.

To better inform the use and development of bromoform feed-additives, this study conducted rumen fluid incubation experiments to: (i) investigate the fate of bromoform in a rumen environment, in order to define a transformation rate and confirm the products; (ii) establish the response of CH_4_ production in the rumen fluid community to varied concentrations of bromoform and its derivatives; (iii) investigate how supplying bromoform, via *Asparagopsis taxiformis* supplementation, influences the rates of transformation and dose-response. Through experiments monitoring the direct incubation of rumen fluid and high-resolution sampling, this work quantifies the dynamics of rapid bromoform transformation and the impact on methanogenesis.

## Materials and methods

### Experimental design and sample collection

Animals used in this study were cared for according to the guidelines of William H. Miner Agricultural Research Institute (Chazy, NY) Animal Care and Use Committee. Rumen fluid was collected by cannulation from four healthy, lactating Holstein cattle maintained on the same diet for five weeks with approval from the William H. Miner Agricultural Research Institute (Chazy, NY) Animal Care and Use Committee and in compliance with SOP#106.

Donor cow profiles and dietary information can be found in supplemental information (Table S1 and Table S2). Rumen samples were strained through four layers of cheesecloth, placed on ice and shipped overnight to Bigelow Laboratory for Ocean Sciences at weekly intervals for three consecutive weeks. Upon arrival, the four samples were combined under anaerobic conditions. Sub-samples of the rumen fluid blend were flash-frozen in liquid nitrogen and stored at −20 °C for later analysis of initial pH, ammonia (NH_3_), and volatile fatty acids (VFAs). The rumen fluid blend was kept air-tight, on ice at 4 °C until used for experimental incubations within four days^34,35^.

### In vitro incubation design and sampling

This study utilized an in vitro technique of directly incubating rumen fluid for short-term experiments (< 6 h). The in vitro experiments were performed in accordance with guidelines reviewed in Yáñez-Ruiz et al.^36^. In order to investigate the response to and transformation of methanogenesis inhibitors, experiments included combinations of the following treatment groups: (i) biotic control (BC): live rumen fluid containing no inhibitors, (ii) biotic plus inhibitor (B+): live rumen fluid containing an inhibitor, (iii) abiotic control plus inhibitor (AC+): rumen fluid sterilized by autoclave (121 °C, 15 psi for 1 h) and containing an inhibitor. Prior to the incubation experiments, 80 ml aliquots of blended rumen fluid were added to 120 ml glass serum bottles and sealed with PTFE septa under anaerobic conditions (80% nitrogen, 15% carbon dioxide, 5% hydrogen). Experiments consisted of a series of 15 to 18 bottles of the same batch of refrigerated rumen fluid incubated in parallel. To revive the microbial activity at the start of each experiment, the incubation bottles, including the killed controls, were brought to 40 °C in a recirculating water bath within 15 min. The bottles thermally equilibrated for another 30 min before the experiment was initiated. The experiment was initiated after headspace sampling (T_0_) and the designated treatment was added. The incubation proceeded for ~ 4–6 h at 40 °C.

**Fig. 1:**
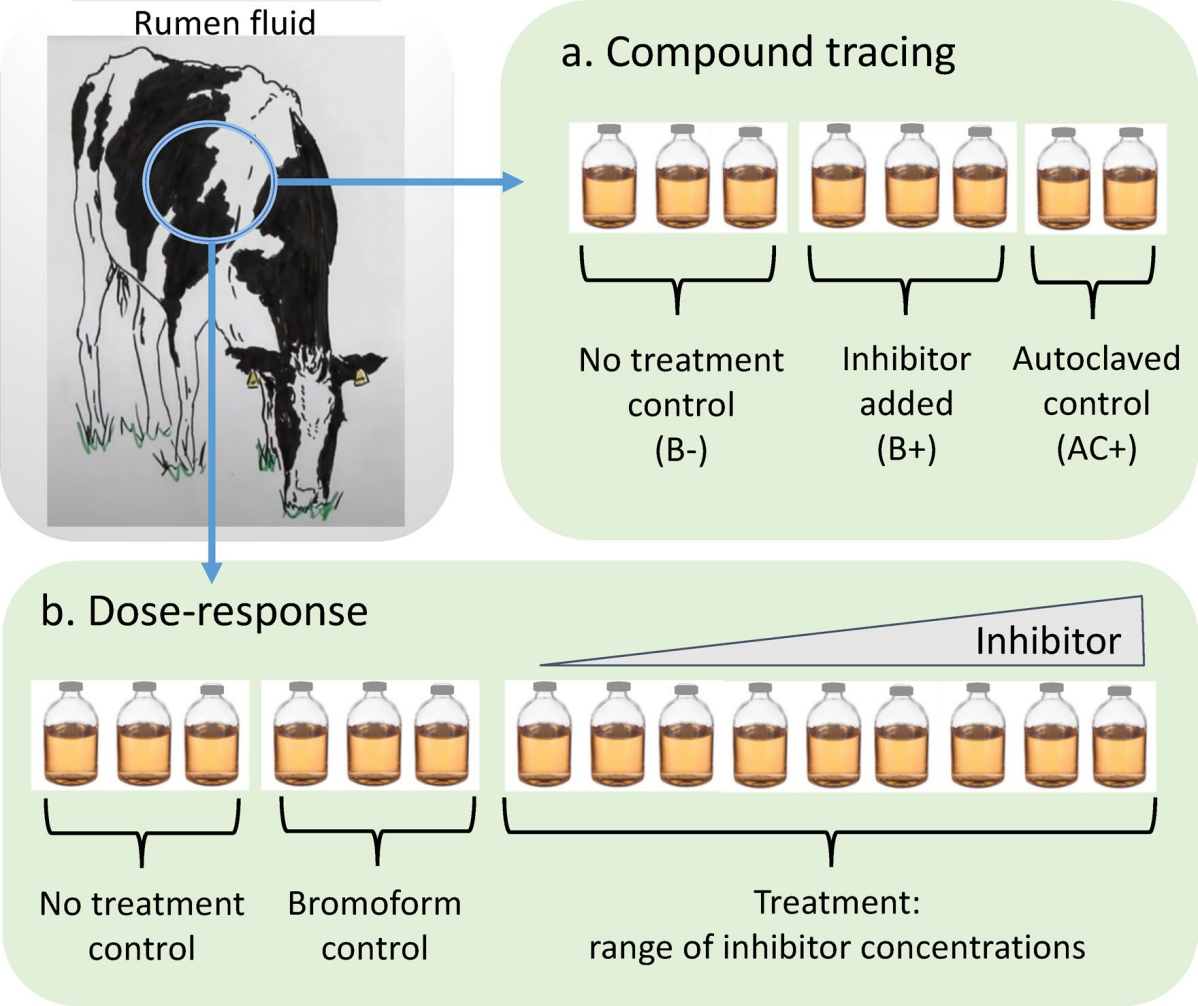
Experimental design of rumen fluid incubations. Weekly shipments of rumen fluid were pooled and used for two different experiment types: (**a**) compound tracing experiments, to investigate the lifetime and transformation of inhibitors; (**b**) dose-response experiments, to determine the sensitivity of methanogenesis to inhibitors.

Two experimental designs were run: i) compound tracing experiments, to investigate the lifetime and transformation of inhibitors; and ii) dose-response experiments, to determine the sensitivity of methanogenesis to inhibitors (Fig. 1). Dose-response experiments included biotic controls (BC), and a series of nine biotic plus inhibitor treatments (B+) across a range of concentrations (0 to 25 µM) containing the specific inhibitor (bromoform (Sigma-Aldrich 99%), dibromomethane (Sigma-Aldrich 99%), bromomethane (Supelco *Trace*CERT^®^), or *Asparagopsis taxiformis*. The *A. taxiformis* was collected fresh from a marine site in the subtropical Atlantic (27° 59’ 19”, −15° 22’ 12”) and stored frozen at −20 °C. The sample material contained 0.33 ± 0.02 mg bromoform g^−1^ dry weight.

Tracing experiment treatments included biotic controls (BC), biotic plus inhibitors (B+), and abiotic plus inhibitors (AC+) of a single concentration of the target compound, each in triplicate. Target compounds bromoform, dibromomethane, and bromomethane, were injected through the septa as solutions in methanol (VWR 99.9%). The corresponding levels of methanol, which ranged from 30 to 950 µl, were added to the control treatments, depending on the desired inhibitor concentration. The *A. taxiformis* was cut into fine pieces, homogenized, pre-weighed into PTFE capsules and placed in the incubation bottles prior to incubation. The amounts of *A. taxiformis* added corresponded to 1 to 4 µmol l^−1^ bromoform, depending on the treatment. The *A. taxiformis* treatment was initiated by puncturing the capsule with a needle to release the seaweed material into the rumen fluid. For tracing experiments, 1.5 ml sub-samples of incubated rumen fluid were taken at approximately 10, 30, 60, 120, 180, and 240 min after addition. For dose-response experiments, sub-samples were taken at 60 and 240 min after addition. Initial and endpoint samples were taken for pH, NH_3_, volatile fatty acids (VFAs), and measurement of dry matter (DM) and were stored at −20 °C.

### Gas measurements and chemical analysis

Headspace samples from the incubations were taken with a gas tight syringe every hour for analysis of the gas volume generated, used to calculate total gas production (TGP); and to determine the CH_4_ concentration, from which CH_4_ production was calculated. To determine CH_4_ concentration, the gas was injected via a 1 ml sample loop on to a gas chromatograph equipped with a thermal conductivity detector (GC-TCD, Shimadzu GC-8 A). The following gas chromatography conditions were used: a Molesieve 13x packed column (60/80 mesh – 2 m) (Restek Corporation), with isothermal oven temperature at 70 °C, a detector temperature of 170 °C and carrier gas flow rate of 50 ml min^−1^. The GC-TCD was calibrated with a CH_4_ analytical standard (99%, MilliporeSigma, 02329).

Quantification of bromoform and dibromomethane in rumen fluid samples was conducted by Bigelow Analytical Services (East Boothbay, ME). A 1 ml aliquot of each rumen fluid sample (liquid) was transferred to a 2 ml glass vial and extracted using 0.5 ml of hexane, containing naphthalene (4 µg ml^−1^) as an internal standard. The samples were vortexed for 60 s, then centrifuged at 5,000 × g for 5 min. The supernatant was transferred to a clean autosampler vial and analyzed on a gas chromatograph with mass-selective detector (Shimadzu GC/MS-QP2010 Ultra). Extraction efficiency for bromoform from rumen fluid was 91 ± 4%. The following gas-chromatography conditions were used: an RTX 502.2 column (30 m x 0.25 mm x 1.4 μm film thickness; Restek Corporation); injection temperature was 200 °C; ion source and interface temperatures were both 220 °C; and the column oven temperature was programmed to start at 40 °C for 4 min, increase at a rate of 15 °C min^−1^ to 80 °C, then increase at 30 °C min^−1^ to a final temperature of 200 °C, and hold for 5 min. Helium was used as carrier gas at a constant pressure of 10.5 psi, giving an initial column flow rate of 1.13 ml min^−1^. For quantification, the major fragment ions were mass/charge ratio (m/z) 173, 174 and 128 for bromoform, dibromomethane and naphthalene, respectively.

The presence of bromomethane in incubation samples was assessed using a purge and trap system coupled to a gas chromatograph with an electron capture detector (GC-ECD)^37^. A 1 ml aliquot of each rumen fluid subsample was placed in a 20 ml glass headspace vial and purged for 10 min with helium gas at a flow rate of 40 ml min^−1^. The analyte was cryo-trapped using liquid nitrogen, and thermally desorbed prior to injection onto the column. Analysis was carried out on a gas chromatograph equipped with electron capture detector (SRI 8610 C GC-ECD, SRI Instruments, USA). The following gas chromatography conditions were used: a ZB-624 column (60 m x 0.32 mm x 1.80 μm film thickness) (Phenomenex); an injection port and valve temperature of 160 °C; a column oven temperature program starting at 30 °C for 10 min, increasing at a rate of 10 °C min^−1^ up to 200 °C, and held for 5 min. Nitrogen was used as carrier gas and make-up gas, at a flow rate of 2.5 and 60 ml min^−1^, respectively. The system was calibrated by making matrix-matched standards using the rumen fluid from each respective experiment and a commercially available bromomethane standard (MilliporeSigma, 48624). The limit of detection was 2 µg l^−1^ of rumen fluid, equivalent to 8 nmol l^−1^.

To determine the bromoform content of the *A. taxiformis*, samples were cut into fine pieces, homogenized by bead beating for 15 min at 30 Hz (Retsch MM 400) and extracted with hexane. Naphthalene was added as an internal standard and samples were analyzed by GC-MS using the approach described above for halocarbon content.

Volatile fatty acids (VFA) samples were stored at −20 ˚C until processing. Samples were thawed on ice and 1 ml aliquots were acidified with 300 µl of 25% phosphoric acid and 100 µl of 20 mmol l^−1^ 4-methylvaleric acid (Aldrich, 277827) (final 1.44 mmol l^−1^) was added as an internal standard. Particulates were removed via centrifugation for 5 min at 27,000 x g at 4 ˚C. The supernatant was filtered through a 0.22 μm 25 mm PTFE syringe filter into 2 ml amber sample vials. Processed samples were analyzed for VFAs by GC-MS (Shimadzu GC/MS-QP2010 Ultra). The following gas chromatography conditions were used: a Nukol™ column (30 m x 0.25 mm x 0.25 μm) (Supelco, 24107); an injector temperature of 200˚C in split mode, with a column flow of helium of 0.71 ml min^−1^, an oven temperature program starting at 100˚C for 1.0 min, increased to 165 ˚C at 5 ˚C min^−1^, followed by an increase to 180 ˚C at 15 ˚C min^−1^, held for 5.0 min. Target compounds were identified and calibrated using a Free Fatty Acid Test Standard (Restek Corporation, 35272).

The pH of the rumen fluid was measured upon receipt, immediately before each experiment, and at the endpoint for each sample/treatment using a pH meter and electrode.

The concentration of NH_3_ in rumen fluid was determined with an NH_3_ ion-selective electrode (Oakton, Cole Palmer). The inner reference solution (0.1 mol l^−1^ NH_4_) and gas-permeable membrane of the electrode were replaced before each use. The electrode was calibrated using a series of NH_4_Cl standards (10, 50, 100, 1000 mg l^−1^). Rumen fluid samples were centrifuged at 3500 x g for 20 min. The supernatant was diluted 10-fold in ultrapure water to a final volume of 50 ml, and 1 ml of 10 N NaOH was added to raise the pH to ≥ 11. The electrode was then immediately immersed in the sample, making sure there were no bubbles on the electrode membrane. Each measurement was stabilized for 4 min before accepting the reading.

Dry matter (DM) was determined by baking 5 ml aliquots of rumen fluid from initial and final time points of the live control treatments of experimental incubations. Triplicate samples were baked at 105˚C to constant mass.

### Calculations and statistical analyses

Total gas production (TGP) was calculated as the gas volume collected from the incubation vial into a glass sampling syringe, under ambient pressure, between time points. This volume was corrected for the changes in headspace volume due to subsampling of the rumen fluid in the vial during the experiments. Methane production was calculated from measured CH_4_ concentration in the expelled gas and the TGP. Dry matter disappearance (DMd) was determined from the difference in initial and final mass of DM in triplicate of the original contents of the donor rumen fluid. VFA concentration was calculated as the sum of the three dominant acids: acetic, propionic and butyric acid. Values of TGP, CH_4_ produced and VFA produced were measured in triplicate incubations unless otherwise stated. Apparent DMd (aDMd), TGP, VFA production and CH_4_ production were normalized to DMd.

To distinguish biological rates of loss for the halogenated compounds from abiotic processes, biotic rates were calculated from the difference between abiotic control incubations (AC+) and the total loss rate observed in the biotic (B+) incubations.

A four-parameter, symmetric log-logistic model was used to describe the results of the dose responses of inhibitor concentration on CH_4_ produced, applied using the *drc* package in R^38,39^. The variables applied in the dose response model were the proportion (%) of CH_4_ produced relative to the average of the three no treatment control incubations and the initial concentration of bromoform (µmol l^−1^) in each treatment incubation (Fig. 1).

## Results

### In vitro rumen fluid characteristics and productivity

Key characteristics of the rumen fluid were measured to verify consistent composition between the batches and between experiments. The initial pH, VFA and DM concentrations varied by less than 20% between batches and remained at similar levels throughout the 24 to 96 h storage periods between receiving the samples and using them in the experiments (Tables 1 and 2).

**Table 1:** Experiment overview. Rumen fluid batches were collected weekly, shipped overnight and stored on ice before being subdivided for the individual experiments. Two types of experiments were performed: compound tracing (T); and dose-response (D). Single inhibitors were added to each experiment: bromoform (BF), dibromomethane (DBM), bromomethane (BM) and *Asparagopsis taxiformis* (*A. Tax*).

Rumen fluid batch	Experiment name	Storage duration (h)	Experiment type	Incubation time (min)	Added inhibitor
1	Exp1	48	T	260	BF
	Exp2	72	T	260	DBM
2	Exp3	48	D	306	*A.Tax*
	Exp4	72	T	260	*A.Tax*
3	Exp5	48	D	285	BF
	Exp6	72	D	240	DBM
	Exp7	96	D	345	BM

**Table 2:** Characteristics of the rumen fluid used for each experiment (Table 1). Data are shown for the unamended incubations. The pH, NH_3_, volatile fatty acid (VFA) and dry matter (DM) content were determined at the start and end of each experiment. Productivity metrics over the 6 to 8 h incubations, are apparent DM disappeared (aDMd) and total gas production (TGP). Production of CH_4_ and VFA is normalized to the quantity of dry matter disappeared (DMd) over the incubation period. The value for VFA is the sum of acetic, propionic and butyric acids. Standard deviations are derived from triplicate incubations.

Experiment	Initial conditions				Final conditions				Productivity			
	pH	NH_3_ (mg N/dl)	VFA (mM)	DM (g)	pH	NH_3_ (mg N/dl)	VFA (mM)	DMd (g)	aDMd (g/g)	TGP/DMd (ml/g)	CH_4_/DMd (mmol/g)	VFA/DMd (mmol/g)
Exp 1	6.81	9.3	92	2.79 ± 0.05	6.11 ± 0.01	41.2 ± 3.4	138 ± 7	0.251 ± 0.003	0.090	363	2.55	14.5
Exp 2	6.84	9.7	104	2.78 ± 0.06	6.03 ± 0.01	44.2 ± 2.2	145 ± 4	0.271 ± 0.004	0.098	340	2.37	11.9
Exp 3	6.64	9.6	113	3.08 ± 0.05	5.82 ± 0.02	44.1 ± 3.7	166 ± 9	0.342 ± 0.004	0.111	368	2.65	12.4
Exp 4	6.63	8.3	97	3.15 ± 0.03	5.86 ± 0.01	38.6 ± 1.8	155 ± 3	0.276 ± 0.003	0.088	341	2.51	16.9
Exp 5	6.31^i^	13.2	-	3.34 ± 0.02	5.53 ± 0.01	39.9 ± 2.4	160 ± 24	0.333 ± 0.012	0.100	348	2.73	-
Exp 6	6.31^i^	13.2	105	3.35 ± 0.02	5.56 ± 0.02	46.4 ± 2.7	157 ± 17	0.265 ± 0.002	0.079	374	2.80	16.7
Exp 7	6.30	12.5	100	3.30 ± 0.03	5.66 ± 0.02	53.9 ± 4.1	160 ± 20	0.342 ± 0.006	0.104	330	2.46	13.2

The largest variation between batches was observed in the NH_3_ concentration of the third batch of rumen fluid but this stayed consistent for each of experiments 5 to 7 (Table 2). Over the three consecutive experimental weeks, rumen fluid collected from the fours cows and incubated without addition of inhibitors (biotic controls), demonstrated similar levels of microbial productivity, including DMd, TGP, changes in VFA and production of CH4, regardless of batch or storage period (Table 2, Figure S1). As expected from an active rumen microbial community, pH levels consistently decreased during the short (~ 4 to 6 h) incubations, as VFA concentrations increased; while NH_3_ levels increased by approximately 4-fold from ~ 10 to ~ 40 mg NH_3_-N/dl in all the incubations (Table 2).

### Rates of bromoform and dibromomethane transformation

**Fig. 2:**
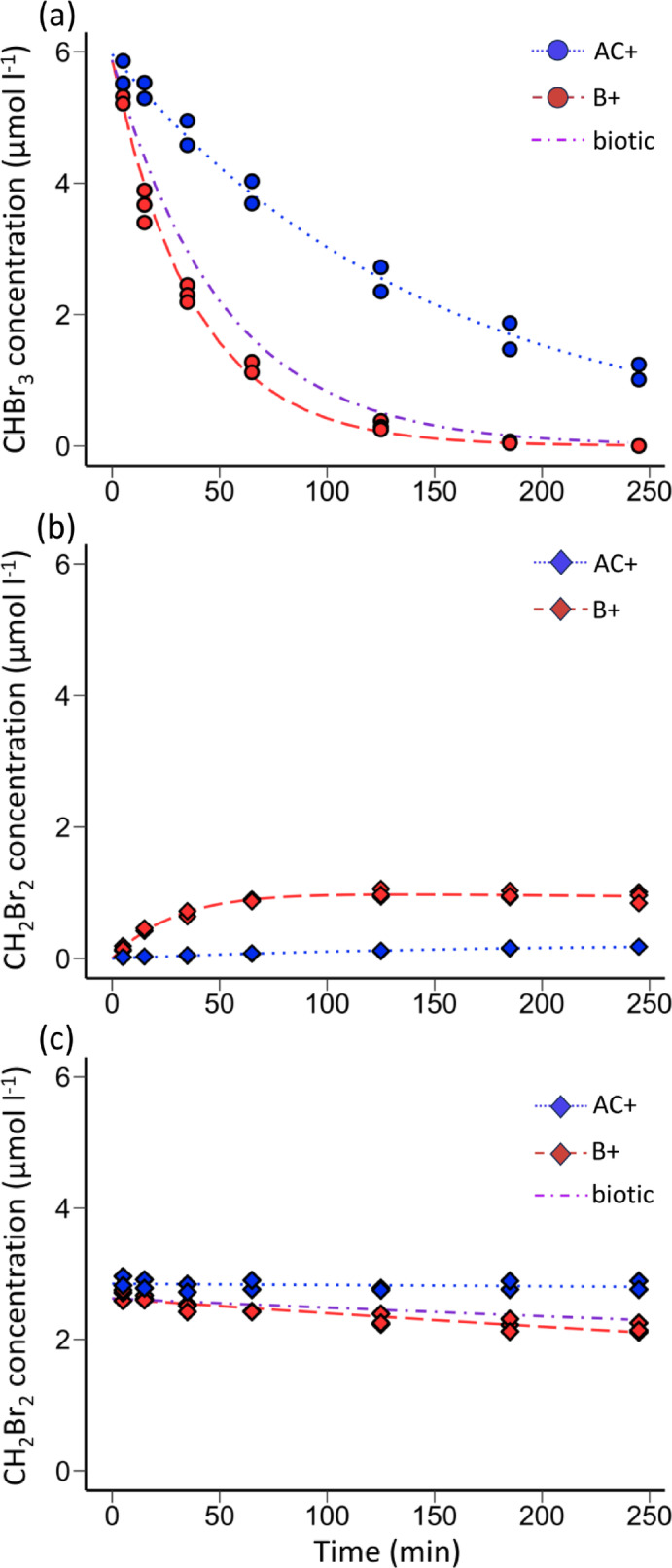
Transformation of bromoform and dibromomethane during in vitro incubations of rumen fluid. (**a**) Bromoform transformation in biotic (B+) and abiotic control (AC+) bromoform amended incubations (Exp1). (**b**) Corresponding dibromomethane concentrations in the B + and AC + incubations to which bromoform had been added (Exp1). (**c**) Dibromomethane concentrations in B + and AC + incubations to which dibromomethane was added (Exp2). Lines represent derived rates, reported in Table 2. Biotic rates were calculated from the differences between total loss (B+, *n* = 3) and abiotic rates (AC+, *n* = 2).

To investigate the persistence of bromoform in rumen fluid, in vitro incubations were dosed and subsampled to monitor concentration over time. In the biotic treatment (B+), bromoform rapidly decreased below detectable levels (Fig. 2A, Exp1). The loss of bromoform occurred at a slower rate in the abiotic control (AC+). The loss of bromoform was associated with the generation of dibromomethane in both B + and AC + treatments (Fig. 2B, Exp1). Dibromomethane began to accumulate immediately as bromoform was degraded, reaching a maximum concentration after 60 min which persisted throughout the incubation of > 240 min (Fig. 2B).

Bromoform degradation was exponential and was dominated by biologically mediated degradation, with loss rates in the live rumen fluid more than 3-fold the loss rates in the autoclaved control incubations (Table 3). When dibromomethane alone was added to rumen fluid, a substantially slower rate of degradation in both biotic and abiotic treatments occurred, compared to bromoform (Fig. 2C, Exp1). The biotic loss of dibromomethane was nearly 30-fold slower than the loss of bromoform, with even lower rates of loss in the abiotic treatments (Table 3). The half-life of bromoform in live rumen fluid was 26 min, compared to 775 min for dibromomethane. The lower degradation rate of dibromomethane favored net accumulation of this derivative in the bromoform addition incubations. In the biotic treatment, dibromomethane accumulation represented approximately 22.1% of the initial bromoform added, compared to 3.4% in the abiotic control (Table 3). Production of monohalogenated bromomethane above a limit of detection of 8 nmol l^−1^, was not detected in any of the incubations.

**Table 3:** Rates of bromoform and dibromomethane transformation in rumen fluid incubations.

Transformation	Rate constant(x 10^-3^min^-1^)	½ life(min)	Yield(%)
Bromoform loss^a^	-26.4 ± 1.3	26	NA
Bromoform abiotic loss^a^	-6.8 ± 0.3	102	NA
Bromoform biological loss^a^	-19.6 ± 1.2	35	NA
Dibromomethane production^b^(µmol l^-1^ min^-1^)	0.0353 ± 0.0024	NA	22.1%
Dibromomethane loss^c^	-0.894 ± 0.091	775	NA
Dibromomethane abiotic loss^c^	-0.00682 ± 0.00685	1.02 x 10^4^	NA
Dibromomethane biological loss^c^	-0.826 ± 0.113	839	NA
Dibromomethane abiotic production^b^ (µmol l^-1^ min^-1^)	0.0014 ± 0.0001	NA	3.4%

The data from which these rates were derived and the corresponding curves are shown in Fig. 2. ^a^Figure 2 A (Exp1)^, b^Figure 2B (Exp1)^, c^Figure 2 C (Exp1).

Based on the Henry’s Law solubility constants for bromoform and dibromomethane, loss to the headspace volume during the incubations would have amounted to < 5% of the dissolved pool of either compound and therefore, had minimal influence on estimated loss and turnover rates. In contrast, approximately 40% of any bromomethane produced during the tracing experiments may have been lost to the headspace gas sampling and may have contributed to the lack of evidence of bromomethane production.

### Methane inhibition dose-response to bromoform, dibromomethane and bromomethane

**Fig. 3:**
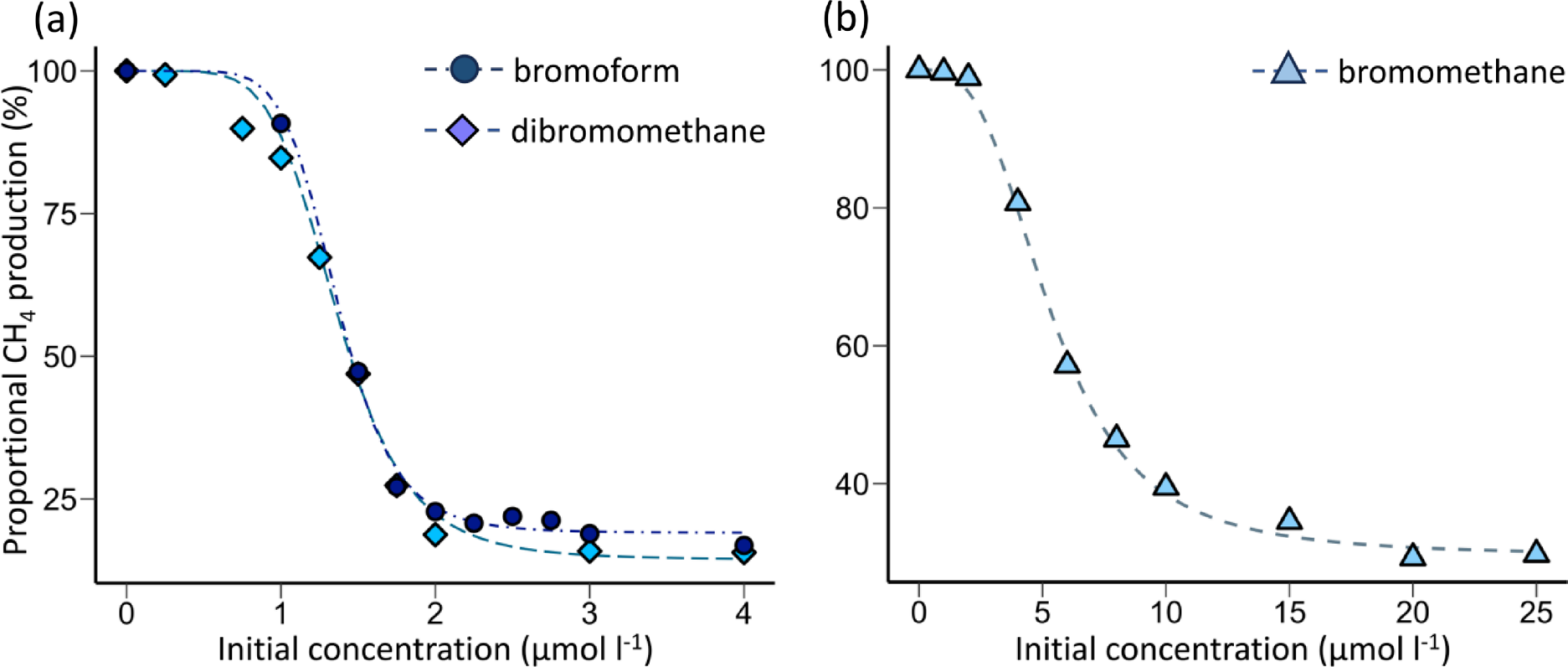
Dose-response of CH_4_ production to bromoform, dibromomethane and bromomethane additions in rumen fluid. The plots show the total CH_4_ produced over 240 min in individual incubations versus the initial rumen fluid concentrations of (**a**) bromoform (Exp5) and dibromomethane (Exp6), and (**b**) bromomethane (Exp7). The four parameters of the log-logistic fits to the data are shown in Table 4. The values of methane production are shown as a proportion of maximum production, which varied slightly between the experiments and is shown as the ‘maximum value’ parameter in Table 4.

Further rumen fluid incubations were conducted to establish a dose-response relationship for bromoform and the potential dehalogenation products dibromomethane and bromomethane. All three brominated compounds demonstrated anti-methanogenic activity and characteristic sigmoidal dose-response curves of CH_4_ production versus inhibitor concentration (Fig. 3). Particularly steep, and very similar, dose responses occurred for bromoform and dibromomethane over a narrow range of 1 to 2 µmol l^−1^ (Fig. 3A). Concentrations > 2 µmol l^−1^ of bromoform or dibromomethane resulted in CH_4_ production of < 20% of the no treatment control, with marginally increased inhibition at higher concentrations. In contrast, the dose response for bromomethane extended over approximately 10 µmol l^−1^, and > 20 µmol l^−1^ of bromomethane was required to achieve a maximal inhibitory effect (Fig. 3B). Bromoform and dibromomethane, despite notable differences in half-life (Table 3), produced nearly identical EC50 values while that of bromomethane was nearly 5-fold higher (Table 4).

**Table 4:** Dose-response relationship of CH_4_ production to bromoform, dibromo- and bromo- methane additions in rumen fluid. The parameters are derived from log-logistic, 4 parameter dose-response curves of cumulative CH_4_ production over 240 min vs. the initial concentration of added inhibitor. Parameter ec-50 represents the effective concentration of the inhibitor resulting in 50 % inhibition of CH_4_ production. The response to each compound was determined on separate days in incubations of the same batch of rumen fluid. The data from which these parameters were derived and the corresponding curves are shown in Figure 3. Uncertainty of the parameters is represented by standard error.

Parameter	bromoform	dibromomethane	bromomethane
coefficient *b*	5.98 ± 0.76	7.46 ± 0.92	3.09 ± 0.24
minimum value (µmol)	98 ± 16	146 ± 8	182 ± 7
maximum value (µmol)	681 ± 14	763 ± 16	616 ± 6
ec-50 (µmol l^-1^)	1.37 ± 0.03	1.36 ± 0.03	5.34 ± 0.14

**Fig. 4:**
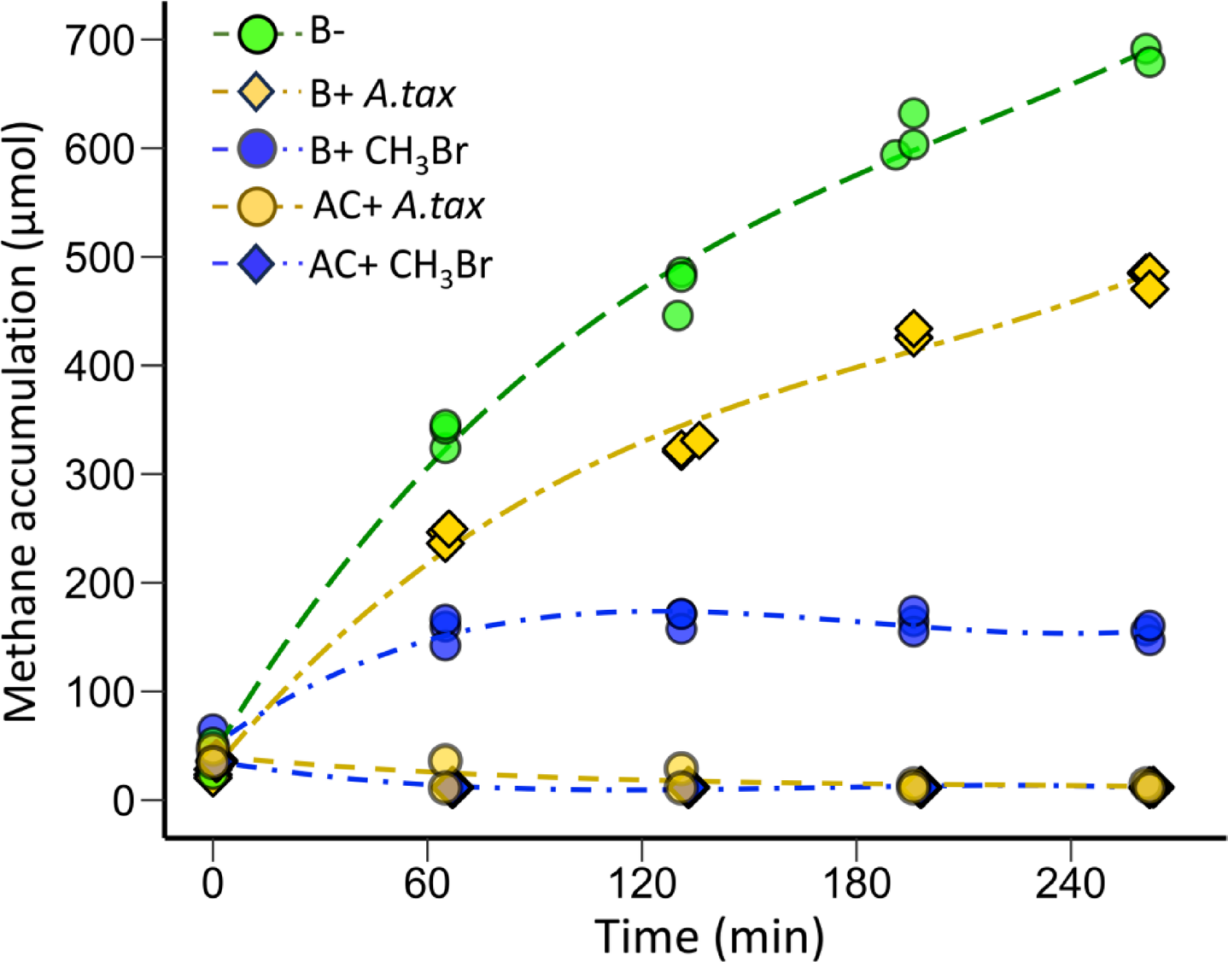
Methane production during incubation of 80 ml of rumen fluid (Exp 4). The treatments were: live rumen fluid with no addition (B-); live rumen fluid with *A. taxiformis*, equivalent to 2.4 µM CHBr_3_ added (B + *A.tax*); bromoform addition resulting in an initial concentration of 3 µM (B + CHBr_3_); and control treatments of autoclaved rumen fluid to which similar levels of bromoform (AC + CHBr_3_) and *A. taxiformis* (AC + *A.tax*) were added.

### Methane response to bromoform and Asparagopsis taxiformis additions

To compare the inhibitory effects of bromoform-containing seaweed versus the pure dissolved compound, *A. taxiformis* was added to rumen fluid incubations. To achieve an inhibitory concentration, *A. taxiformis* corresponding to 2.4 µmol l^−1^ bromoform was added in the B + *A.tax* treatments. The biotic plus inhibitor treatment (B + CHBr_3_) significantly reduced CH_4_ production compared to the control within ~ 60 min of addition (Fig. 4). The *A. taxiformis* treatment (B + *A.tax*) also resulted in lower CH_4_ production than the control incubation, which persisted throughout the incubation (Fig. 4). However, the *A. taxiformis* incubation displayed less CH_4_ inhibition than the bromoform alone. Specifically, the *A. taxiformis* treatment containing 2.4 µmol l^−1^ bromoform resulted in 31% inhibition of the maximum CH_4_ production in the biotic control (B-) after 260 min, compared to the bromoform treatment (B + CHBr_3_) of 3 µmol l^−1^, which produced 78% CH_4_ inhibition over the same time period. Although the analyzed bromoform concentration differed slightly between the two treatments, the dose-response for pure bromoform (Fig. 3) indicated that both the bromoform (B + CHBr_3_) and (B + *A.tax*) treatments should have produced similar levels of CH_4_ inhibition.

**Fig. 5:**
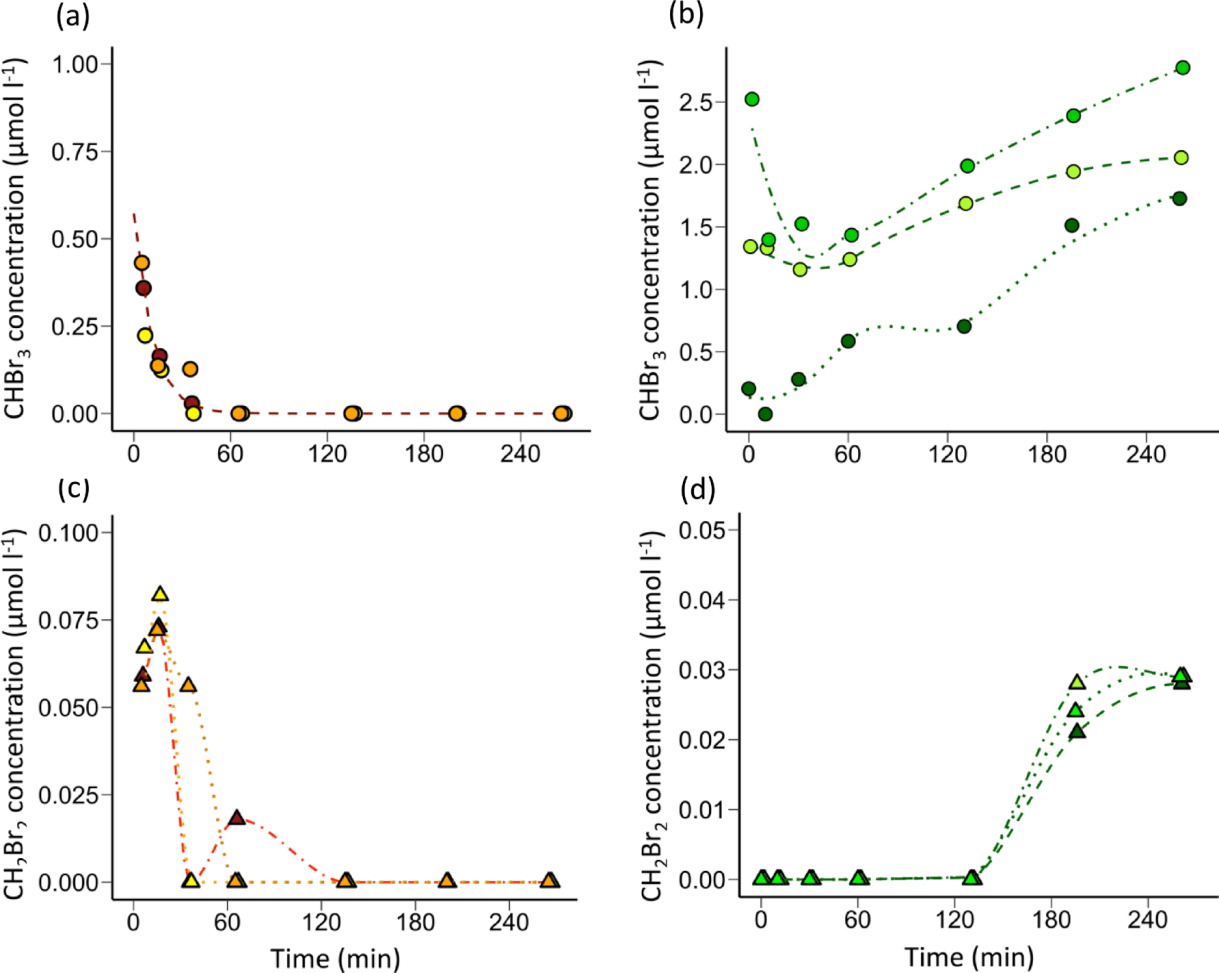
Release and transformation of bromoform following *A. taxiformi*s addition to rumen fluid (Exp 4). The results are from three replicate incubations for each treatment, shown as different symbols and lines. (**a**) Bromoform concentrations in the dissolved phase of biotic rumen fluid, in three ‘replicate’ incubations following addition of a similar mass of *A. taxiformis*. (**b**) Bromoform in the dissolved phase of abiotic, autoclaved rumen fluid, following addition of similar amounts of *A. taxiformis*. (**c**) Dibromomethane in the same biotic rumen fluid incubations to which *A. taxiformis* had been added. (**d**) Dibromomethane in the same abiotic, autoclaved rumen fluid. Note the limit of detection for dibromomethane was 0.02 µmol l^−1^. Hence, points shown as 0 µmol l^−1^ may have been underestimates of the true concentration.

**Fig. 6:**
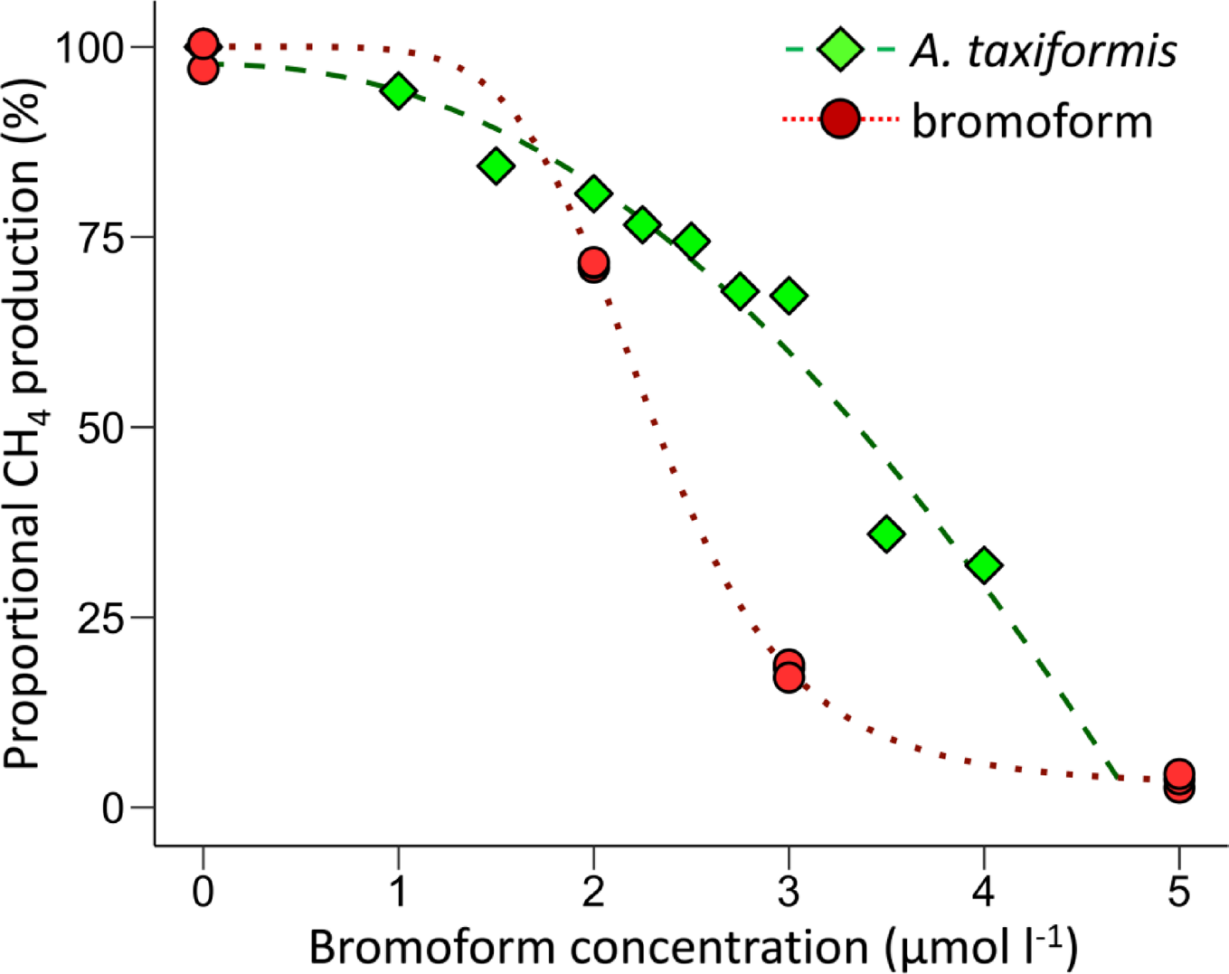
*Asparagopsis taxiformis* dose-response on CH_4_ production from in vitro rumen fluid incubations (Exp 3). Shown is the proportional CH_4_ production over ~ 300 min in individual incubations with nine varied additions of *A. taxiformis* and three levels of dissolved bromoform addition. The values of CH_4_ production are shown as a proportion of maximum production (100% CH_4_ production), determined in triplicate incubations of the biotic control to which no inhibitor was added.

### Asparagopsis taxiformis – derived bromoform tracing and dose-response

To understand the fate of bromoform derived from *A. taxiformis*, bromoform and dibromomethane concentrations were monitored in rumen fluid incubations as in previous experiments. The initial dissolved bromoform concentration in the rumen fluid was significantly lower than predicted from the added amounts of *A. taxiformis*. In the biotic plus inhibitor treatment (B + *A.tax*), the maximum dissolved concentration measured was 0.34 µmol l^−1^, equivalent to 14% of the total bromoform content in the added seaweed (Fig. 5a). The majority of the bromoform presumably initially remained in particles of *A. taxiformis* and was released at a slower rate than degradation occurred, preventing an accumulation in the dissolved phase. Although initial dissolved bromoform concentrations varied between triplicate incubations, the dissolved bromoform concentration increased at similar rates after 30 min in the abiotic treatment (Fig. 5b). The rate of release of bromoform to the dissolved phase from the added seaweed exceeded the degradation rate in the abiotic treatment, resulting in net accumulation. Similar to the bromoform addition experiments, the formation of dibromomethane was observed in both the biotic and abiotic incubations. The peak levels of dibromomethane coincided with the rapid degradation of bromoform in the biotic treatment, reaching 0.076 ± 0.006 µmol l^−1^ or 22.0% of the highest observed bromoform (Fig. 5c). In the abiotic incubation 0.030 ± 0.001 µmol l^−1^ dibromomethane was observed at the end of the incubation (Fig. 5d), equivalent to 1.3% of the maximum bromoform concentrations (Fig. 5b).

In the dose-response experiment, nine levels of *A. taxiformis* addition, equivalent to total bromoform concentrations in the rumen fluid of 1 µmol l^−1^ to 4 µmol l^−1^, were monitored for CH_4_ production. These levels of *A. taxiformis*-derived bromoform were compared to three dissolved bromoform treatments of a similar concentration range (Fig. 6). In contrast to the dissolved bromoform additions, increasing levels of *A. taxiformis* produced a less rapid decrease in CH_4_ production, that did not follow the characteristic sigmoidal relationship observed for the dissolved additions (Figs. 3A and 6). Methane inhibition was observed across the range tested, however, *A. taxiformis* additions equivalent to the dissolved bromoform additions produced less of a response. For example, incubations to which *A. taxiformis* addition resulted in a total bromoform addition of 3 µmol l^−1^, generated only ~ 25% inhibition of CH_4_ production compared to > 75% inhibition from an equivalent amount of dissolved bromoform (Fig. 6). The delayed release of bromoform into the dissolved phase in the *A. taxiformis* addition treatments, most evident in the abiotic incubations (Fig. 5B), results in a reduced inhibition of CH_4_ production compared to similar addition levels of free bromoform (Fig. 6).

## Discussion

### Rumen fluid incubations

The experimental approach of the present study was designed to examine specific questions regarding the inhibition of CH_4_ production and degradation rates of bromoform in a rumen system over short time scales. Using proven practices of rumen fluid preservation^34,35^, in vitro methodology^36^ and short incubations (< 6 h), these experiments aimed to closely approximate in vivo levels of rumen activity. The approach contrasts with commonly used approaches involving the inoculation of buffered media with rumen fluid and longer incubation times^40,41^. Nonetheless, the consistent results between experiments (Table 1) and as will be discussed, the close alignment to in vivo rates, support the use of rumen fluid preserved by refrigeration for short-duration incubations of the type used in the present study.

Although direct comparisons between metabolic rates of in vitro and in vivo rumen microbial consortia are challenging (e.g., Macome et al.^42^), a number of metrics illustrate close simulation of in vivo rumen function in the in vitro incubations^42^. Initial VFA concentrations of the incubated rumen fluid varied little (92 to 105 mM) over the 4-week experimental study and remained within a range typical of diurnal variations in in vivo concentrations of 80 to 180 mM^43^, over the course of each experiment (Table 1). Increasing VFA concentrations during the incubations are reflected in the decreases in pH between initial and final time points (Table 1), that varied by similar levels to the diurnal range of pH of 5.5 to 6.8, observed in freshly sampled rumen fluid or by using indwelling pH sensors in lactating dairy cows^44,45^. The range of NH_3_ concentrations in the batches of refrigerated rumen fluid averaged 10.7 to 41.7 mg-N dl^−1^ (7.6 to 29.8 mmol l^−1^) at initial and final time points of the incubations (Table 1), typical of reported in vivo rumen levels of 1 to 23 mmol l^−1^^46^. The 4-fold increases in NH_3_ during the ~ 6 h incubations (Table 1) are similar to those observed in refrigerated samples by Fabro et al.^34^, who attributed it to enhanced protein metabolism stimulated by the cold storage. Although, similar levels of increase of 5 to 25 mg-N l^−1^, have been observed in the rumen shortly after feed intake^47^. Comparisons of CH_4_ production between in vitro and in vivo systems are informative when the gas production can be normalized to similar parameters of substrate utilization. For instance, respiration chamber estimates of CH_4_ production in lactating Holstein Friesian dairy cows varied between 1.95 and 2.15 mmol g^−1^ of digested organic matter, when fed diets that varied in starch content^48^. Comparable CH_4_ production estimates of 2.37 to 2.80 mmol g^−1^ DMd, were observed in the in vitro incubations (Table 1). These similarities in trends in VFA, pH, NH_3_ and CH_4_ production between the in vitro incubations and in vivo observations, support the validity of the rumen fluid incubation approach of the present study.

Nonetheless, the observations made in the present study should be interpreted as a snapshot of the rumen state and the rates of halogenated compound loss and production pertain only to rumen fluid-mediated processes at a certain phase of digestion. In common with alternative in vitro approaches, the measurements potentially differ from in vivo rates due to oxygen exposure during sample collection, exclusion of mat- and epithelium-associated microbial communities; and for this specific approach, cryo-storage-induced artifacts. Moreover, the estimated rates do not account for loss processes that would occur in an animal, including during oral-esophageal passage to the rumen, absorption by rumen epithelium, or solid, fluid and gas export from the rumen.

### Processes of bromoform transformation

The rapid degradation of bromoform, with a half-life of ~ 26 min at the levels examined (< 5 µM, Table 3), confirms that it is unlikely to accumulate in the rumen or be transferred to rumen-derived feed products when administered at minimum effective levels^28^. However, the formation of dibromomethane, which had a significantly longer half-life of 775 min in the incubations (Table 3), suggests accumulation and persistence of bromoform degradation products is possible, potentially long enough to be absorbed by the animal or to pass through the digestive system. The degradation of bromoform and subsequent production of dibromomethane was previously reported in an in vitro study using a buffered rumen batch culture incubated over longer incubation times of up to 72 h^33^. Although faster initial loss of bromoform in the buffered rumen batch cultures of Romero et al.^33^ precludes straightforward comparisons to the rates obtained in the present study, dibromomethane was produced in a comparable proportion of 20 to 40% of the degraded bromoform. The rapid biological degradation of bromoform and relative recalcitrance of dibromomethane in the rumen fluid (Fig. 2) resembles the reductive dehalogenation of chloroform by the methanogen *Methanosarcina barkeri*^24^. Metabolism of chloroform by *M. barkeri* produced very little chloromethane and no detectable CH_4_. If sequential dehalogenation of bromoform and dibromomethane in the rumen fluid had occurred it would have produced bromomethane and the terminal products CH_4_ and bromide. While, this study was not designed to resolve bromoform-derived CH_4_ from the bulk CH_4_ production and attempts to detect bromide in the rumen fluid were confounded by matrix interferences, no detectable bromomethane production resulted from the degradation of bromoform. *In vivo s*tudies have demonstrated an increase in bromine content in milk, blood, urine, feces, suggesting further breakdown is likely^49^. However, when dibromomethane alone, was added to abiotic or biotic rumen fluid, rates of loss were low, indicating limited transformation to other compounds (Fig. 2C; Table 3). Hence, the gradual build-up of dibromomethane from bromoform dehalogenation (Fig. 2B), is unlikely to be due to a depletion of Coenzyme F430 degradation activity, as suggested by Romero et al.^33^, but more likely due to the relatively low yields of transformation from bromoform.

Reductive dehalogenation by methanogens or heterotrophic anaerobes may not account for all the biotic loss of bromoform in the rumen. Other members of the microbial community could also be responsible for bromoform degradation. Both acetogenic and sulfate-reducing bacteria occur in the rumen and, in addition to methanogens, are capable of dehalogenation of aliphatic halogens, primarily through co-metabolic processes^50,51^.

Although rates of bromoform loss were higher in the biotic treatments, significant loss of bromoform occurred in the abiotic treatment (Fig. 2A). This resembles the dehalogenation activity reported in heat-killed cultures of three anaerobic bacteria, which was attributed to the catalytic activity of heat-stable corrinoid cofactors^50^. However, there are several other abiotic processes that could have contributed to the dehalogenation rates and may occur in the rumen, including base-catalyzed hydrolysis of halomethanes^52,53^ and nucleophilic substitution. It is possible that chlorinated compounds, generated through substitution and non-volatile halogenated compounds were formed in the breakdown of bromoform, but would not have been captured in the present study^54^.

More research is required on the controls of rumen dehalogenation and the impact of sustained additive feeding on the microbial populations. Prolonged exposure to bromoform and similar compounds could alter microbial composition in the rumen, particularly within the methanogen community and alter the capacity for their transformation^12,55^. In vivo, factors such as feed intake rates and timing and the residence time of the additive in the rumen could influence the levels of impact on the microbial composition.

### Implications of rapid bromoform loss and dose-response relationship

The degradation of bromoform and transformation into the antimethanogenic derivative dibromomethane provides insight into the dynamics of CH_4_ inhibition. A decrease in CH_4_ production following bromoform addition was observed in < 60 min (Fig. 3). The rapid loss of bromoform (Fig. 2A), indicates that inhibition of CH_4_ production likely commenced almost immediately. The inhibitory impact persisted after bromoform was degraded to levels below the inhibition threshold of ~ 1 µmol l^−1^ (Fig. 2), suggesting prolonged effects on the metabolic function and/or community abundance of the methanogens. Although *A. taxiformis* is reported to produce a variety of halogenated compounds, bromoform is the only one produced in sufficient concentrations to be an inhibitor of methanogenesis at the levels of seaweed added in this study^12,13^. The formation and accumulation of dibromomethane likely contributed to the antimethanogenic efficacy of bromoform.

The halogenated compounds evaluated in this study exhibited dose-response relationships over a narrow concentration range of 1 to 2 µmol l^−1^ for bromoform and dibromomethane and 10 µmol l^−1^ for bromomethane (Fig. 3). This suggests that in the case of bromoform, achieving in-rumen concentrations of close to 2 µmol l^−1^ would result in near maximum inhibition of enteric CH_4_ production. However, multiple factors contribute to determining the actual bromoform concentration to which methanogens would be exposed in vivo. As the slightly more gradual response curve for *A. taxiformis*-bromoform demonstrates (Fig. 6), the form in which the bromoform is delivered to the rumen microbial community may influence the concentration to which methanogens are exposed and the CH_4_ inhibition response. Nonetheless, animal trials in which treatments have included varied amounts of *A. taxiformis* fed over prolonged time scales, also demonstrate a narrow range of sensitivity between CH_4_ emission and bromoform administered^6,8,9,21^. A compilation of five animal trials that have fed varied amounts of *A. taxiformis*, demonstrated an inhibition yield of −0.34 g CH_4_ mg^−1^ bromoform ingested^14^. Comparable values for the in vitro incubations of the present study in which *A. taxiformis* was added in varying amounts (Fig. 6), averaged − 0.96 g CH_4_ mg^−1^ bromoform addition. The increased sensitivity of the in vitro system could be caused by several factors including: (i) the direct addition of bromoform to the rumen fluid, bypassing potential losses in the feed mix and during ingestion by the animals; (ii) the relatively short incubation period of this study relative to the longer time-integrated nature of the in vivo trials; and (iii) diel variability in the in-rumen bromoform concentrations as a result of animal behavior.

## Conclusion

The short duration incubations of cold-preserved rumen fluid provided reproducible and informative quantification of the potency and fate of bromoform and derivatives, including when added in the form of *A. taxiformis* tissue. The results demonstrate the potential of developing halogenated feed additives that accurately deliver the target concentrations of inhibitor to cause methanogenic inhibition and minimize both absorption by the animal of excess halogenated compounds, particularly dibromomethane, and limit their transfer to the environment. Additives comprising preserved *Asparagopsis* spp. have been successfully applied at near-optimum levels in a series of animal trials. Lower in vivo yields of CH_4_ inhibition versus level of bromoform dosing compared to the in vitro assays may be due to the slower release of bromoform from *Asparagopsis* spp. tissue illustrated in the present study but are most likely due to additional losses of bromoform during feeding and while in the digestive system. Few of the animal trials carried out to date involving bromoform-containing feed additives, appear to have tested for dibromomethane in animal residues or milk^56^. An exception is the recent study by Williams et al.^49^ that fed different dietary formulations of *A. armata* to lactating Holstein-Friesian cows and did measure higher concentrations of dibromomethane than bromoform in the urine and blood, confirming the findings of the present study^49^. New formulations of halogenated feed additives clearly need to adequately monitor the fate of their potential breakdown products. Understanding the systemic response to bromoform inhibition could benefit from modeling approaches that transform in vitro information of the type generated in the present study to predictions of the on-farm situation.

## Electronic supplementary material

Below is the link to the electronic supplementary material.

Supplementary Material 1

## Data Availability

All data generated and/or analyzed during the current study are available from the corresponding author on reasonable request.

## Abbreviations

NH_3_: Ammonia
aDMd: Apparent dry matter disappeared
Br-: Bromide
CH_4_: Methane
CHBr_3_: Bromoform
CH_2_Br_2_: Dibromomethane
CO_2_: Carbon dioxide
DM: Dry matter
DMd: Dry matter disappearance
DMI: Dry matter intake
EC50: 50% effective concentration
GC-ECD: Gas chromatograph – electron capture detector
Mcr: Methyl-coenzyme M reductase
Mtr: Coenzyme M methyltransferase complex
PTFE: Polytetrafluoroethylene (Teflon)
THMs: Trihalomethanes
TGP: Total gas production
VFA: Volatile fatty acids
