# Supplementary material for: The antimethanogenic efficacy and fate of bromoform and its transformation products in rumen fluid
Source: Sci Rep. 2025 Jul 11;15:25171. doi: 10.1038/s41598-025-10936-9 (PMC12254416; doi:10.1038/s41598-025-10936-9)
Supplement: Supplementary file 1 — Supplementary Material 1 [file 41598_2025_10936_MOESM1_ESM.docx]

**Supplemental Material**


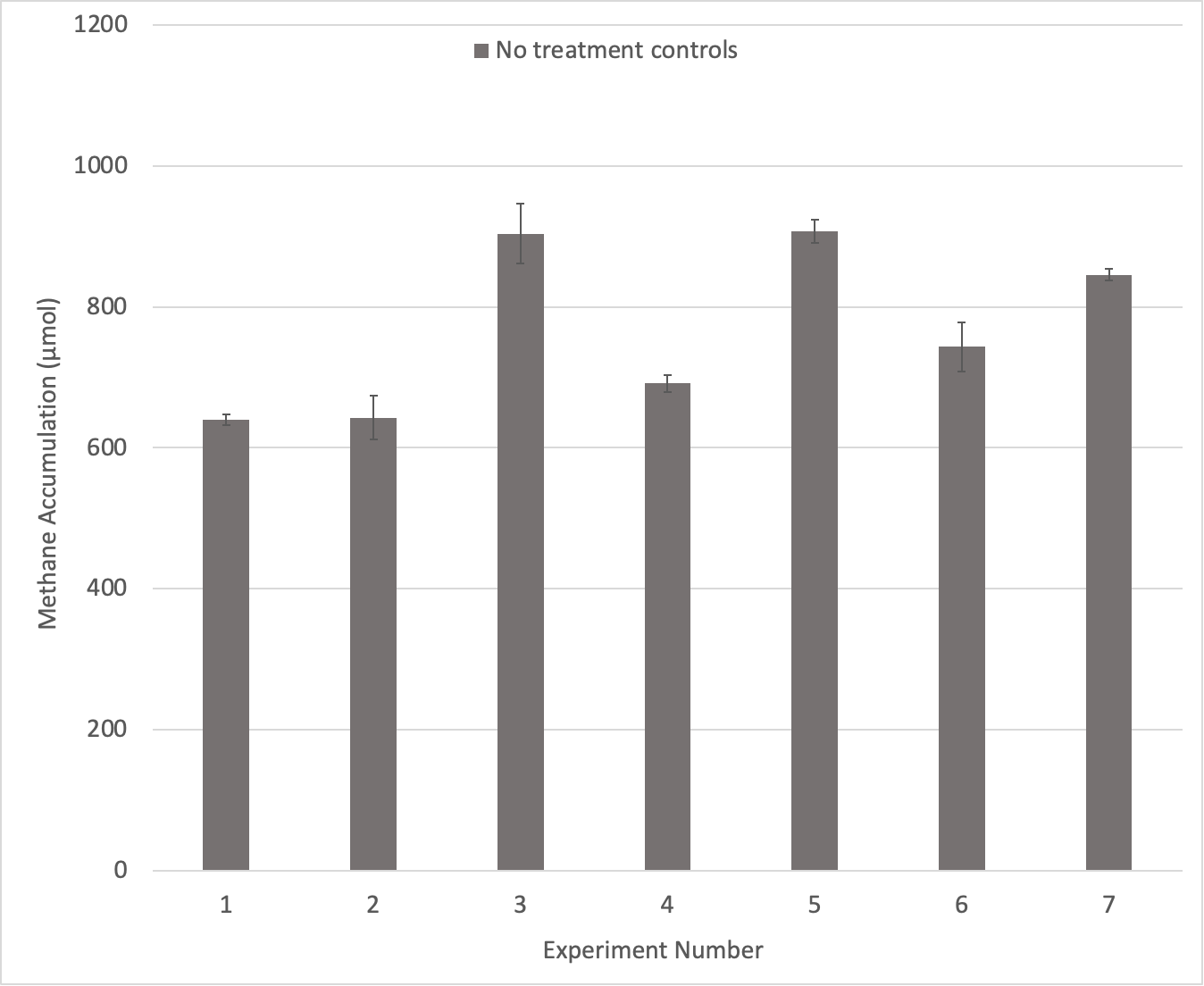


**Figure S1**. Total methane production by biotic controls for all experiments.

| **Donor Cow ID** | **Milk production average and SD (kg)** | **Days in Milk** |
| --- | --- | --- |
| 3427 | 38.6 ± 0.9 | 301 to 329 |
| 3448 | 40.9 ± 0.6 | 257 to 285 |
| 3404 | 32.7 ± 0.7 | 286 to 314 |
| 3239 | 44.3 ± 0.7 | 244 to 272 |

**Table S1**. Donor cow milk production. The data represents milk production over the period corresponding with weekly shipments of rumen fluid from Feb 28th, 2022 to Mar 28^th^, 2022.

| **Output** | **Value** |
| --- | --- |
| Dry Matter Intake. (lbs/day) | 58.75 |
| DM (%) | 50.69 |
| Forage (%DM) | 52.34 |
| aNDFom (%DM) | 27.85 |
| Value Sugar (%DM) | 5.56 |
| Starch (%DM) | 26.14 |
| RUFAL Intake (g) | 799.52 |

**Table S2**. Donor cow diet. The data represents the diet maintained over the period corresponding with weekly shipments of rumen fluid from Feb 28th, 2022 to Mar 28^th^, 2022.
